# Vancomycin Dosing Regimen to Obtain the Target Area Under the Concentration–Time Curve, Which Provides an Early Treatment Response for Patients on Haemodialysis

**DOI:** 10.3390/antibiotics15010047

**Published:** 2026-01-03

**Authors:** Minori Kambe, Takashi Ueda, Kazutaka Oda, Kazuhiro Sugiyama, Kazuhiko Nakajima, Naruhito Otani, Motoi Uchino, Yuki Horio, Ryuichi Kuwahara, Masanobu Toyama, Makoto Tomita, Atsuki Ide, Mayuko Ao, Yasuhiro Nozaki, Yoshio Takesue

**Affiliations:** 1 Department of Nephrology, Chita Peninsula General Medical Organization, Rinku Hospital, 3-3-3 Asukadai, Tokoname 479-8510, Aichi, Japan; kambeminori611@gmail.com (M.K.); sugihiro@fj9.so-net.ne.jp (K.S.); mtomitam1961@yahoo.co.jp (M.T.); mikeandryo@yahoo.co.jp (A.I.); kymrsamnossnsd7@icloud.com (M.A.); 2 Department of Infection Prevention and Control, Hyogo Medical University Hospital, 1-1 Mukogawa-cho, Nishinomiya 663-8501, Hyogo, Japan; taka76@hyo-med.ac.jp (T.U.); nakajima@hyo-med.ac.jp (K.N.); n-otani@hyo-med.ac.jp (N.O.); 3 Department of Pharmacy, Kumamoto University Hospital, 1-1-1 Honjo, Chuo-ku, Kumamoto 860-8556, Japan; kazutakaoda1980@gmail.com; 4 Division of Inflammatory Bowel Disease Surgery, Department of Gastroenterological Surgery, Hyogo Medical University, 1-1 Mukogawa-cho, Nishinomiya 663-8501, Hyogo, Japan; uchino2s@hyo-med.ac.jp (M.U.); yu-horio@hyo-med.ac.jp (Y.H.); ri-kuwahara@hyo-med.ac.jp (R.K.); 5 Pharmaceutical Department, Chita Peninsula General Medical Organization, Rinku Hospital, 3-3-3 Asukadai, Tokoname 479-8510, Aichi, Japan; ma-1080da@outlook.jp; 6 Department of Respiratory Medicine, Chita Peninsula General Medical Organization, Rinku Hospital, 3-3-3 Asukadai, Tokoname 479-8510, Aichi, Japan; goblue0239@gmail.com; 7 Department of Infection Prevention and Control, Chita Peninsula General Medical Organization, Rinku Hospital, 3-3-3 Asukadai, Tokoname 479-8510, Aichi, Japan

**Keywords:** vancomycin, haemodialysis, area under the concentration–time curve, early treatment response, MRSA infection

## Abstract

**Objectives**: This study aimed to clarify the area under the curve (AUC) for obtaining better clinical outcomes and to demonstrate vancomycin dosing for achieving the AUC in haemodialysis (HD). **Methods**: The vancomycin concentration was measured before the second HD. The AUC_24–48h_ after the initial HD was assessed to evaluate its correlation with an early clinical response and to determine the dosing regimen, assuming an inter-dialysis interval of 48 h, even if the interval was 72 h. **Results**: An AUC/MIC ≥ 400 was an independent factor for an early response in treating MRSA infections and infections caused by methicillin-resistant Gram-positive organisms. An AUC of 600–700 μg·h/mL did not increase the incidence of adverse effects compared with that of <600 μg·h/mL. An AUC of 400–700 μg·h/mL was obtained in 90.5% of patients with a loading dose of 30 mg/kg followed by a maintenance dose of 10 mg/kg. Pre-dialysis concentrations were significantly higher than the trough concentration required in non-HD patients to achieve the same AUC category, and AUC_24–48h_ was strongly correlated with pre-dialysis concentrations (R^2^ = 0.921). In a receiver operating characteristic curve, the cut-off value for an early response was 16.8 μg/mL for the pre-dialysis concentration/MIC. **Conclusions**: AUC_24–48h_ after the initial HD/MIC of ≥400 μg/mL improves the clinical outcomes in patients on HD, and the target PK/PD can be achieved with an upper range of the recommended dose. The pre-dialysis concentration may be a reliable surrogate for the AUC, and the vancomycin dose could be adjusted according to this PK target.

## 1. Introduction

*Staphylococcus aureus*, including methicillin-resistant *Staphylococcus aureus* (MRSA), is the primary cause of vascular access infection and bloodstream infection in patients on haemodialysis (HD) [1] because of the high MRSA nasal colonization rate [2] and frequent access to the bloodstream. The Centers for Disease Control and Prevention reported that patients on HD were 100 times more likely to have bacteraemia by *S. aureus* than the general population [3]. Vancomycin is the first choice of antibiotics for treating MRSA infection. Physicians are usually not concerned about therapeutic drug monitoring (TDM) for vancomycin in patients receiving HD because nephrotoxicity is not a major issue for this population. However, because MRSA bacteraemia has a five-fold higher risk of death in patients on HD than in patients not on HD [4], a vancomycin dosing strategy to obtain better treatment outcomes should be prioritized.

Area under the curve (AUC)-based dosing is recommended for vancomycin in patients who are not on dialysis [5,6,7]. Software for Bayesian estimation developed with the collaboration of the Japanese Society of Chemotherapy and Japanese therapeutic drug monitoring is available for free of charge. Therefore, AUC-based doing for vancomycin is widely used in Japan [8]. However, evaluation of the AUC in patients on HD is complicated because several factors, such as dialyser permeability, plasma and dialysate flow rates, and drug-related factors, are required to predict it [9]. Additionally, because of variability of the elapsed time to the first HD session after vancomycin administration and an inconsistent inter-dialysis interval (i.e., 48 or 78 h in thrice-weekly HD), the day 1 or day 2 AUC, which is commonly used as a pharmacokinetic (PK) parameter in patients without HD [10], cannot be applied to patients on HD. There is a substantial difference in the PKs of vancomycin between patients with and without HD. Consequently, the target AUC in patients on HD should not be simply extrapolated from the AUC recommended in patients with normal renal function without confirmation with a clinical study.

This study aimed to clarify the AUC/minimum inhibitory concentration (MIC) to improve clinical outcomes. We also aimed to provide a better understanding of vancomycin dosing to achieve the target AUC and a corresponding TDM strategy using the pre-dialysis concentration in a thrice-weekly HD setting.

## 2. Results

**Figure 1:**
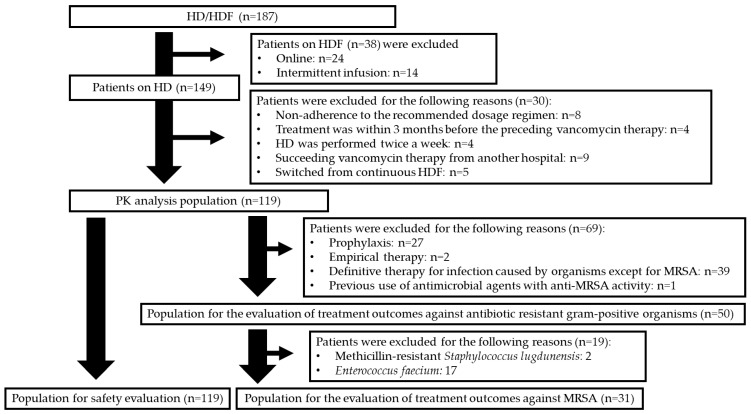
A flow chart of the patients’ selection. HD = haemodialysis; HDF = haemodiafiltration; PK = pharmacokinetic.

A total of 119 patients were eligible for PK and safety analyses (Figure 1). Treatment outcomes were investigated in 31 patients with MRSA infections and 50 patients infected with MRSA, *Enterococcus faecium*, or methicillin-resistant *Staphylococcus lugdunensis*.

HD-related factors are shown in Table S1. Polysulfone, which is a high-flux membrane, was used as a haemodialyser in most patients. The mean duration of the HD session was 3.5 ± 0.5 h, and the mean blood flow rate was 174.6 ± 36.7 mL/min. TDM was conducted 5.6 ± 1.1 days after initiating vancomycin. The mean elapsed time to the first HD session after starting vancomycin was 39.3 ± 4.0 h. An early clinical response and an early onset of adverse effects were assessed at 5.1 ± 0.7 days. The mean duration of vancomycin therapy was 17.6 ± 14.8 days. MICs of vancomycin against MRSA were 0.5 μg/mL in 2 strains, 1.0 μg/mL in 28 strains, and 2.0 μg/mL in 1 strain.

The period between the first and second HD sessions was divided into two or three 24 h terms according to the inter-dialysis interval (48 or 72 h). The median AUC 0–24 h after the first HD (AUC1st _term_) and median AUC 24–48 h after the initial HD (AUC2nd _term_) was 454.1 μg·h/mL (interquartile range [IQR]: 407.6–519.1) and 444.2 μg·h/mL (391.0–501.6), respectively, in all eligible 119 patients for PK analysis (*p* < 0.001). In 37 patients with an inter-dialysis interval of 72 h, AUC1st _term_, AUC 2nd _term_, and the AUC 48–72 h after the initial HD (AUC3rd _term_) was 491.5 μg·h/mL (442.5–521.9), 468.6 μg·h/mL (408.7–505.4), and 422.1 μg·h/mL (371.1–458.8), respectively (*p* < 0.001).

Using the AUC2nd _term_, a significantly higher early clinical response rate against MRSA infections was observed in patients with an AUC2nd _term_/MIC ≥ 400 than in those with an AUC2nd _term_/MIC < 400 (78.9% versus 25.0%, *p* = 0.003) (Table 1). Similarly, when patients with resistant Gram-positive organisms were evaluated collectively, the early clinical response rate was also higher in those with an AUC2nd _term_/MIC ≥ 400 than in those with an AUC2nd _term_/MIC < 400 (*p* < 0.001) (Table 1). There were no significant differences in the incidence of hepatotoxicity, neutropenia, thrombocytopenia, or overall myelosuppression between patients with an AUC2nd _term_ of 600–700 μg·h/mL and those with an AUC ≤ 600 μg·h/mL (Table 2). Although ototoxicity was not evaluated in nine unconscious patients and in one patient with pre-existing hearing disturbance, none complained of ototoxicity among 109 evaluable patients. An AUC2nd _term_ ≥ 700 μg·h/mL was not experienced in any patients. On the basis of these results, the upper threshold of the AUC2nd _term_ was determined as 700 μg·h/mL in this study.

Taken together, these findings suggested that the initial dosing regimen could be targeted to achieve an AUC2nd _term_ of 400–700 μg·h/mL in patients on HD. In multivariate analyses, an AUC2nd _term_/MIC ≥ 400 (adjusted odds ratio [OR]: 23.14; 95% confidence interval [CI]: 2.31–431.53) and the achievement of source control were independent factors for an increased early clinical response in patients infected with MRSA (Table 3) However, the extremely wide CI caused by a small sample size indicated model instability and inadequate events per variable. An AUC2nd _term_/MIC ≥ 400 (adjusted OR: 24.04; 95% CI: 3.831–151.08) was also an independent factor for an increased early clinical response in patients infected with resistant Gram-positive infections (Table S2).

The mean initial two cumulative doses was 28.5 ± 1.8 mg/kg in patients with the low-dose regimen, 35.8 ± 1.8 mg/kg in the standard-dose regimen, and 40.6 ± 1.5 mg/kg in the high-dose regimen. The achievement rate of the target AUC2nd _term_ was 0%, 69.1%, and 90.5% in the three dose regimens, respectively (Table 4). On the basis of the loading dose (30.7 ± 1.3 mg/kg) and the maintenance dose (9.9 ± 0.7 mg/kg) in the high-dose regimen (Table 4), a single dose of 30 mg/kg followed by 10 mg/kg after each session was suggested to obtain the target AUC2nd _term_.

**Figure 2:**
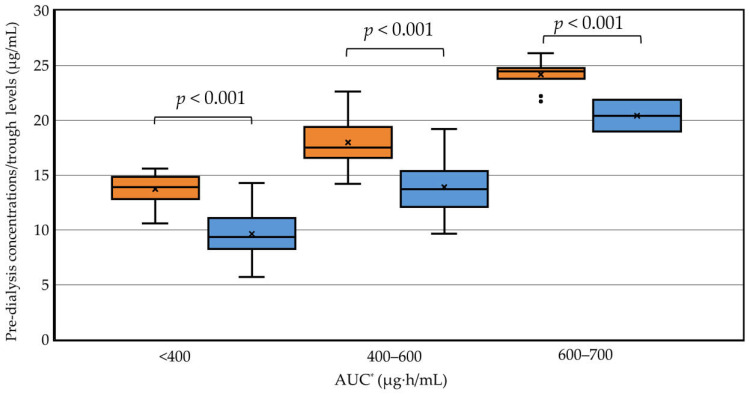
Pre-dialysis concentrations in patients on HD and trough concentrations after the fourth dose in patients without HD according to AUC categories. * The AUC _24–48h_ after the initial HD session (AUC2nd _term_) was used in patients on HD, and the day 2 AUC (AUC_24–48h_) after starting vancomycin therapy was used in patients not on HD. Orange boxplots show patients on HD and blue boxplots show patients not on HD.

The pre-dialysis concentration before the second HD session and the trough concentration in patients without HD were compared according to the AUC categories. The background of patients on HD compared with that of patients without HD is shown in Table S3. The AUC on day 2 was predicted with two-point sampling in 77 of 112 patients without HD. The AUC2nd _term_ was used in patients on HD. The pre-dialysis concentration was significantly higher than the trough concentration in each AUC category (*p* < 0.001 in each) (Figure 2). An AUC of 400–600 μg·h/mL was obtained with a median pre-dialysis concentration of 17.5 μg/mL (IQR: 16.6–19.4) and a median trough concentration of 13.7 μg/mL (IQR: 12.2–15.3).

**Figure 3:**
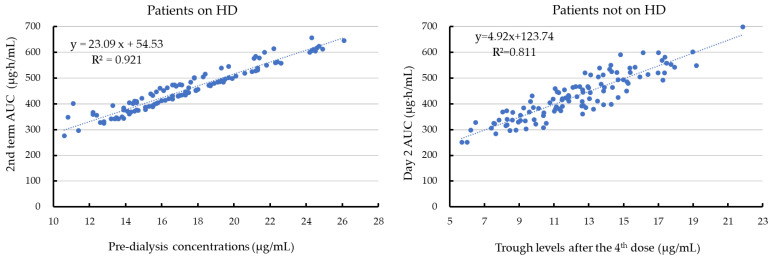
A scatter diagram of the relationship between the pre-dialysis concentration and the AUC2nd _term_ in patients on haemodialysis (HD) and that between the trough concentration after the fourth dose and the day 2 AUC (AUC_24–48h_) after starting vancomycin therapy in patients not on HD.

A scatter diagram of the relationship between the pre-dialysis concentration/trough concentration and the AUC is shown in Figure 3. In the regression analysis, there was a strong correlation between the AUC2nd _term_ and pre-dialysis concentration (R^2^ = 0.921). In contrast, the scatter diagram of the relationship between the trough concentration and the AUC on day 2 after starting therapy in patients without HD showed a significantly weaker correlation than that between the AUC2nd _term_ and pre-dialysis concentration (*p* = 0.001). However, there was no significant difference in the correlation coefficient between patients with and without HD, when the AUC1st _term_ was used for the relationship with the pre-dialysis concentration in patients on HD.

**Figure 4:**
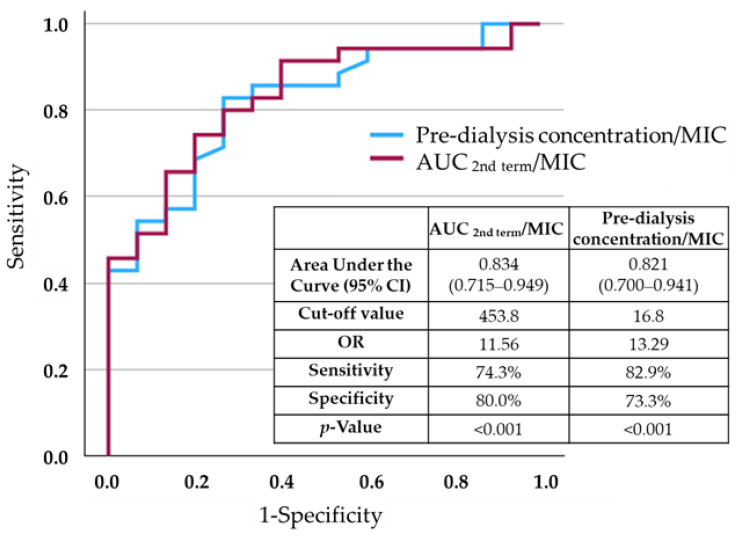
A receiver operating characteristic curve for discriminating the early clinical response in patients infected with resistant Gram-positive organisms using the AUC2nd _term_/MIC ratio or pre-dialysis concentration/MIC ratio.

In the receiver operating characteristic (ROC) curve analysis of an early clinical response in patients with MRSA infections, the area under the curve was 0.744 for the AUC2nd _term_/MIC ratio and 0.739 for the pre-dialysis concentration/MIC ratio. In patients infected with resistant Gram-positive organisms, area under the ROC curve was 0.834 for the AUC2nd _term_/MIC ratio and 0.821 for the pre-dialysis concentration/MIC ratio. In these patients, there was a higher discrimination ability for an early clinical response than in those with MRSA infections. The cut-off values were 453.8 for the AUC2nd _term_/MIC ratio and 16.8 for the pre-dialysis concentration/MIC ratio (Figure 4).

## 3. Discussion

In this study, the ratio of the AUC2nd _term_ to an MIC ≥ 400 was an independent factor for an early clinical response with vancomycin therapy against MRSA infections and infection with resistant Gram-positive organisms under the conditions of a high-flux haemodialyser, low blood flow rate setting, and post-dialysis vancomycin administration. The upper 24 h AUC limit of 600 μg·h/mL associated with nephrotoxicity in patients without dialysis is less concerning in patients with end-stage kidney disease requiring HD. We found that the AUC2nd _term_ could be increased up to 700 μg·h/mL without increasing adverse effects, including hepatotoxicity, myelosuppression, and ototoxicity. Additionally, the dosing regimen required to obtain a target AUC2nd _term_ of 400–700 μg·h/mL was a loading dose of 30 mg/kg followed by a maintenance dose of 10 mg/kg after each HD session.

Using Monte Carlo simulation, Lewis et al. predicted that post-dialytic administration of a loading dose of 25 mg/kg and a maintenance dose of 10 mg/kg are most likely to obtain an AUC_24h_ ≥ 400 mg·h/L in patients on high-flux HD [11]. Polášková et al. [12] stated that loading doses of 2250 and 2750 mg should be administered to patients with obesity and a lean body mass of 70–80 kg and >85 kg, respectively. While AUC targets have been validated in patients without HD, whether the same targets apply to patients on HD is unclear. In studies that used Monte Carlo simulation for patients on HD, the dose was generally determined to meet an AUC ≥ 400 mg·h/L, which has been recommended for patients not requiring HD [11,12,13,14]. Therefore, the primary aim of this study was to determine a target AUC that would cause beneficial effects for clinical outcomes without increasing the risk of detrimental effects. After defining the target AUC range in a clinical study, we attempted to determine the vancomycin dose required to achieve the PK target.

Variable elapsed times to the first dialysis session after starting vancomycin or an inconsistent inter-dialysis interval in a thrice-weekly HD setting (48 h twice and 72 h once per week) make analyses difficult for clinical studies. In this study, we did not divide the eligible patient population into two groups according to the interval because of the decreasing evaluable number of patients in each group over time. To resolve this issue, the AUC2nd _term_ was used to determine the dosing regimen to achieve the target AUC, with the assumption that the inter-dialysis interval was constant at 48 h. Consequently, the dosing regimen proposed in this study should be adjusted in clinical practice to account for a longer interval [15]. In a 72 h interdialytic period, a 30% higher maintenance dose or an additional 250 mg is required to maintain target attainment [11,16]. An increase in the loading dose is also required for a prolonged elapsed time to the initial HD session. A loading dose of 35 mg/kg or an additional dose of 1 g 24 h after the loading dose has been proposed if HD is delayed by 72 h [17,18].

Regarding determining the AUC to obtain beneficial clinical outcomes, the AUC in the last term would have been the best PK target for the evaluation of clinical outcomes in our study. This time would have been best because attainment of the target PK in the last 24 h term assures a sufficient concentration throughout the inter-dialysis interval. However, the AUC2nd _term_ used to determine vancomycin dosing should also be assessed for the PK/pharmacodynamic (PD) target to increase the clinical response. The time–concentration curves in the second and third terms correspond to the elimination phase. Therefore, in this study, a mild decrease was anticipated during these terms in patients on HD, and the difference between AUC2nd _term_ and AUC3rd _term_ was only 10.6% (468.6 μg·h/mL and 422.1 μg·h/mL, respectively).

The pre-dialysis concentration is considered to be a good surrogate to the AUC compared with the trough concentration in patients not on HD. In this study, the pre-dialysis concentration/MIC ratio was significantly correlated with an early clinical response, and the area under the ROC curve was 0.821 and the cut-off value was 16.8 μg·h/mL. Similar to our results, Fu et al. reported that a pre-dialysis concentration/MIC ratio of ≥18.6 was associated with the eradication of MRSA by vancomycin therapy in patients on HD [19]. In our study, the pre-dialysis concentration was significantly higher than the trough concentration to achieve the same AUC category in patients without HD. Higher pre-dialysis concentrations are recommended in the guidelines not only because of the negligible adverse effects, including acute kidney injury, but also to improve clinical outcomes [20,21].

This study has several limitations. First, all AUC values were simulated from a single concentration point without validation. Therefore, the reported strong correlation between the AUC and pre-dialysis concentration could have been a modelling artifact rather than a true PK relationship. Second, the dosing recommendations were based on post hoc simulations rather than measured exposures and should be interpreted cautiously. Third, factors that are unique in Japanese HD practice should be considered in the application of our results to global patients [22]. Japanese practice guidelines recommend that HD should involve a long treatment duration and a low blood flow rate (approximately half that used in the USA), and Kt/V_urea_ values should remain <1.2 [22]. Therefore, a high-performance membrane, which effectively removes β-2-microgloblin, is used as the standard haemodialyser in Japan [23]. In contrast to Japan, many dialysis centres in the USA and Europe administer vancomycin during dialysis (1 h before the end of HD), especially for outpatient treatment. Approximately 20–30% of the vancomycin dose infused during dialysis is subject to dialytic removal. Therefore, a 30% larger vancomycin dose should be considered if infused intra-dialytically, and an increased loading dose (35 mg/kg) and maintenance dose (15 mg/kg) in high-flux HD are encouraged [11].

Fourth, the cut-off pre-dialysis concentration for an early clinical response was determined in infections caused by resistant Gram-positive organisms because of a relatively low number of patients with MRSA infections. The use of a higher concentration than the cut-off value used in this study would be required in the treatment of MRSA infections. Fifth, PK analysis and a dose recommendation were not performed for haemodiafiltration (HDF). Predilution online HDF is widely used in Japan because of the difficulty in substituting a sufficient volume caused by the low blood flow rate [24]. Other reasons include the reduction of albumin loss and the suppression of membrane fouling during treatment. In contrast to post-dilution treatment, adequate clinical evidence that predilution online HDF to provide a better outcome of patients has not been reported. In addition, the number of patients who have intermittent infusion HDF dialysis to prevent a rapid drop in blood pressure during a dialysis session and to improve peripheral circulation is increasing in Japan [25]. Sixth, a greater number of patients is required for evaluating the safety of an AUC > 600 μg·h/mL in patients on HD. Seventh, because no patients had follow-up audiometry, ototoxicity was possibly overlooked. Finally, this was a retrospective study. Therefore, our findings may not be generalizable because of a lack of sufficient evidence.

## 4. Materials and Methods

### 4.1. Ethics

Approval for this study was obtained by the Ethics Committee of Hyogo University Hospital (202508-066). The board waived the requirement for informed consent from the patients included in this study and an opt-out approach was used. Patients on HD and patients without HD as a control group admitted to Hyogo Medical College and Chita Peninsula General Medical Organization Rinku Hospital between January 2021 and March 2025 were retrospectively included in the study.

### 4.2. Inclusion and Exclusion Criteria for Patients on HD

The inclusion criteria for the PK or safety analysis population were as follows. (1) Patients in whom at least three doses of vancomycin were administered with the dosage recommended in clinical practice guidelines were included [5,26]. The loading dose was 20–25 or 25–30 mg/kg followed by a maintenance dose of 7.5–10 mg/kg immediately after each HD session. A dose of 10–12 mg/kg was also added in accordance with a recent report [9]. (2) Patients underwent HD scheduled thrice weekly. (3) A serum sample was taken within 1 h before the second HD session, and vancomycin concentrations were measured using a commercial reagent kit (Vanc Flex; Siemens Healthcare Diagnostics, Tokyo, Japan). The coefficient of the dynamic range was 0.8–50 μg/mL.

The exclusion criteria were as follows: (1) <15 years old; (2) patients treated with HDF; (3) vancomycin administration during HD; (4) patients treated by vancomycin within 3 months; (5) patients with succeeding vancomycin therapy from another institution; (6) patients who switched from continuous HDF; and (7) unexpected survival for 5 days. Treatment outcomes, including an early clinical response, treatment success at the end of therapy, and 30-day mortality, were primarily evaluated in patients with MRSA, and secondarily in those with resistant Gram-positive organisms, including MRSA, *Enterococcus faecium*, and methicillin-resistant *Staphylococcus lugdunensis*.

### 4.3. Classification of the Vancomycin Dosing Regimen

The initial two cumulative doses, including a loading dose and a maintenance dose, were administered as follows. The standard-dose regimen was 35 mg/kg (31.5–38.5 mg/kg with rounding), the low-dose regimen was 27.5 mg/kg (24.75–31.5 mg/kg), and the high-dose regimen was 42 mg/kg (38.5–46.2 mg/kg). In addition, the loading dose and the maintenance dose were classified individually. The loading dose was administered as follows: (1) standard dose, 25 mg/kg (22.5–27.5 mg/kg with rounding); (2) low dose, 20 mg/kg (18–22.5 mg/kg); and (3) high dose, 30 mg/kg (27.5–33.0 mg/kg). The maintenance dose was as follows: (1) standard dose, 10 mg/kg (9.0–11.0 mg/kg with rounding); (2) low dose, 7.5 mg/kg (6.75–9.0 mg/kg); and (3) high dose, up to 12 mg/kg (11.0–12.0 mg/kg).

### 4.4. Definitions of Adverse Effects and Clinical Efficacy

Adverse effects were evaluated at 5 ± 1 days and at the end of therapy. Hepatotoxicity was defined as ≥Grade 1 in the Common Terminology Criteria for Adverse Events v. 5.0. Leukopenia was defined as a total peripheral white blood cell count <4 × 10^9^/L and thrombocytopenia as a reduction in the platelet count to <75%. Overall myelosuppression including anaemia, which was defined as 2 g/dL reduction in haemoglobin concentrations was also evaluated [27]. The presence of ototoxicity was determined from the medical records in conscious patients without pre-existing hearing loss.

An early clinical response at 5 ± 1 days and treatment success at the end of therapy were evaluated. Patients were defined as early clinical responders if they had a 30% decrease in the total white blood cell count or C-reactive protein concentrations, a decline in fever in febrile patients (defined as a daily decease of >0.3 °C for at least 2 consecutive days), no worsening of clinical features, and survival for ≥96 h [28]. Treatment success was defined as survival with resolution or improvement of all core symptoms, signs, laboratory data, and radiographic abnormalities caused by infections, and further therapy by agents with anti-MRSA activity was unnecessary.

### 4.5. Estimating the AUC over a Specified Period in Patients on HD

The AUC for a specified period was estimated using the arithmetic mean of simulated drug concentrations over that period, multiplied by the corresponding elapsed time. PK simulations were conducted on the basis of individual parameters estimated by Bayesian inference, using measured vancomycin concentrations. Bayesian estimation was performed using custom-developed software (https://bmspod.web.fc2.com/, written in Japanese; accessed on 1 August 2025), which operates via Visual Basic for Applications within Microsoft^®^ Excel. Concentration values at each time point for plotting the concentration–time profile were calculated numerically using the Runge–Kutta–Gill method.

The population PK model (Table S4), along with the algorithm used to estimate extracorporeal clearance for a drug by HD, was adopted [9]. Required parameters included plasma and dialysate flow rates, as well as the ultrafiltration rate, depending on the membrane performance, which was characterized by baseline clearance values for creatinine and vitamin B12 (molecular weight, 1355 Da, which is similar to that of vancomycin [1449 Da]). During HD sessions, extracorporeal clearance was transiently added to the patient’s physiological (body) clearance.

### 4.6. Evaluation of the AUC

**Figure 5:**
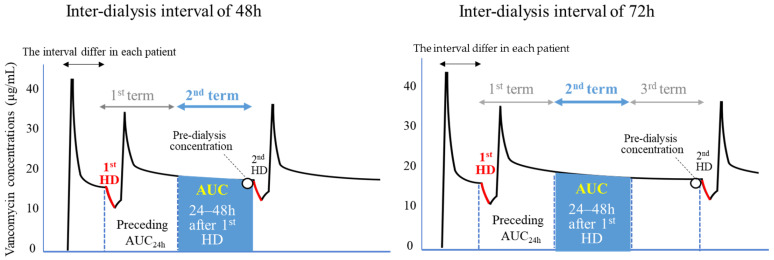
The AUC 24–48 h after initial HD was used as a pharmacokinetic parameter affecting treatment outcomes and adverse effects and to determine the dosing regimen. The assumption that the inter-dialysis interval was 48 h even in patients with an interval of 72 h was made. TDM = therapeutic drug monitoring; HD = haemodialysis.

The PKs for the first maintenance dose combined with a loading dose were studied between the first and second HD sessions during which two or three 24 h terms were included according to the inter-dialysis interval (two terms in an inter-dialysis interval of 48 h and three in an interval of 72 h) (Figure 5). Because the AUC1st _term_ after the initial HD session included an HD session, peak level, and distribution phase, a low prediction accuracy was anticipated for the AUC1st _term_. The AUC2nd _term_ (24–48 h after the first HD session) divided by the MIC was assessed for the relationship with clinical outcomes. The dose regimen to obtain the target PK range defined by the AUC2nd _term_ was evaluated, with the assumption that the interval is 48 h irrespective of the inter-dialysis interval (48 or 72 h). The MIC of vancomycin was measured using microdilution methods in accordance with the Clinical and Laboratory Standards Institute testing guidelines (M02 and M07, 2018) [29].

### 4.7. Relationship Between Trough Concentrations and AUC_24h_ in Patients Without HD

The PKs of vancomycin in patients without HD were analysed using the Bayesian estimation software PAT, which was developed in the R program (https://www.r-project.org/) as the platform using the r-shiny package [30]. A previously reported Japanese population PK model was used for the a priori probability [8]. The timing of blood sampling for the trough concentration was inconsistent in patients without HD. Therefore, the Bayesian-predicted concentration 11 h after the end of the fourth infusion in patients with vancomycin q12h administration was used. The relationship between the predicted trough concentration and the AUC on day 2 (24–48 h after starting vancomycin) was examined in patients without HD.

### 4.8. Statistical Methods

Continuous variables are presented as the mean ± standard deviation if the data followed a symmetric distribution. The median (IQR) was used if the data were skewed because the mean value can be distorted by outliers. Parametric variables were analysed using Student’s *t*-test, whereas nonparametric variables were analysed using the Mann–Whitney U-test. ROC curves were used to identify cut-off values of PK/PD parameters for an early clinical response.

Multivariate analyses were performed to determine the adjusted OR for factors associated with nephrotoxicity. A univariate analysis was performed to estimate each variable using the chi-square test, and potential confounders were examined using cross-tabulation. Variables selected in the univariate analysis (*p* < 0.2) were subsequently entered into a stepwise logistic regression model to estimate the magnitude of association (adjusted OR and 95% CI). The level of significance was set at *p* < 0.05. SPSS ver. 30 (IBM Corp., Armonk, NY, USA) was used to perform the analyses.

## 5. Conclusions

This study shows that an AUC_24h_/MIC ratio ≥ 400, which is recommended in patients without HD, is an independent factor for an increased early clinical response to vancomycin therapy against MRSA infections and infection with resistant Gram-positive organisms in patients on HD. An increase up to the upper threshold of the vancomycin dosing method recommended for patients on HD in the guidelines is required to meet the AUC_24h_ targets [20]. Dosing adjustment based on a single vancomycin serum concentration obtained before the second HD session is recommended. However, because of the associative nature of our findings, a prospective validation to clarify how the dosing regimen and corresponding TDM could be integrated into a real-world strategy for optimal vancomycin therapy in patients on HD is required.

## Figures and Tables

**Table 1:** Treatment outcomes in patients on haemodialysis infected with MRSA and those with resistant Gram-positive organisms based on the cut-off ratio (400) of the AUC2nd _term_ to the MIC.

Population for Treatment Outcome Evaluation	Treatment Outcomes	AUC2nd _term_/MIC < 400 No. of Patients (%)	AUC2nd _term_/MIC ≥ 400 No. of Patients (%)	*p* Value
Patients infected with MRSA (n = 31)	Early clinical response	3/12 (25.0)	15/19 (78.9)	0.003
	Clinical success at the end of therapy	7/12 (58.3)	18/19 (94.7)	0.022
	30-day mortality	3/12 (25.0)	0/19 (0.0)	0.049
Patients infected with resistant Gram-positive organisms (n = 50)	Early clinical response	3/12 (25.0)	32/38 (84.2)	<0.001
	Clinical success at the end of therapy	7/12 (58.3)	37/38 (97.4)	0.002
	30-day mortality	3/12 (25.0)	1/38 (2.6)	0.038

**Table 2:** Incidence of adverse effects according to the AUC2nd _term_.

Adverse Effect	Observation Period	AUC2nd _term_		*p* Value
		<600 μg·h/mL (n = 109)	600–700 μg·h/mL (n = 10)	
Hepatotoxicity, no. of patients (%)	On day 5 (±1 day)	13 (11.9)	2 (20.0)	0.613
	At the end of therapy	17 (15.6)	2 (20.0)	0.660
Neutropenia, No. of patients (%)	On day 5 (±1 day)	1 (0.9)	0 (0.0)	1.000
	At the end of therapy	4 (3.7)	1 (10.0)	0.360
Thrombocytopenia, No. of patients (%)	On day 5 (±1 day)	6 (5.5)	0 (0.0)	1.000
	At the end of therapy	11 (10.1)	1 (10.0)	1.000
Myelosuppression, no of patients (%)	On day 5 (±1 day)	7 (6.4)	0 (0.0)	1.000
	At the end of therapy	14 (12.8)	2 (20.0)	0.623
Ototoxicity, no. of patients (%)	At the end of therapy *	0 ^†^ (0)	0 (0)	–

* An interview regarding ototoxicity was not obtained on day 5 in many patients. Therefore, only the result at the end of therapy is shown. ^†^ Ten patients were excluded because of unconsciousness or pre-existing hearing loss.

**Table 3:** Univariate and multivariate analyses of variables associated with an early clinical response in patients with MRSA infection.

Factor	No. of Patients with an Early Clinical Response (%)		Univariate Analysis		Multivariate Analysis	
	Patients with a Particular Factor	Patients Without a Particular Factor	Crude OR (95% CI)	*p* Value	Adjusted OR (95% CI)	*p* Value
Male sex	13/21 (61.9)	5/10 (50.0)	1.63 (0.36–7.43)	0.701		
ICU stay	2/4 (50.0)	16/27 (59.3)	0.69 (0.08–5.64)	1.000		
Age ≥ 65 years	16/25 (64.0)	2/6 (33.3)	3.56 (0.54–23.39)	0.208		
Body mass index < 18.5 kg/m^2^	3/7 (42.9)	15/24 (62.5)	0.45 (0.08–2.49)	0.413		
Body mass index ≥ 25 kg/m^2^	5/9 (55.6)	13/22 (59.1)	0.87 (0.18–4.14)	1.000		
Surgery within 30 days	4/6 (66.7)	14/25 (56.0)	1.57 (0.24–10.22)	1.000		
Severity of illness						
SOFA score > 5 (median)	7/13 (53.8)	11/18 (61.1)	0.74 (0.18–3.15)	0.686		
Septic shock	0/0	18/31 (58.1)	–	–		
Mechanical ventilation	1/2 (50.0)	17/29 (58.6)	0.71 (0.04–12.43)	1.000		
Type of infection						
Complicated by MRSA infection *	8/17 (47.1)	10/14 (71.4)	0.36 (12.08–1.59)	0.171	0.34 (0.07–1.65)	0.079
Bloodstream infection	3/6 (50.0)	15/25 (60.0)	0.67 (0.11–3.99)	0.676		
Bone and joint infection	7/12 (58.3)	11/19 (57.9)	1.02 (0.24–4.41)	0.981		
Skin and soft tissue infection	6/9 (66.7)	12/22 (54.5)	1.67 (0.33–8.42)	0.696		
Respiratory tract infection	5/8 (62.5)	13/23 (56.5)	1.28 (0.25–6.69)	1.000		
Intra-abdominal infection	0/0	18/31 (58.1)	–	–		
Urinary tract infection	0/0	18/31 (58.1)	–	–		
Co-infection with Gram-negative bacteria	10/16 (62.5)	8/15 (53.3)	1.46 (0.35–6.11)	0.605		
Co-infection with a fungal infection	0/0	18/31 (58.1)	–	–		
AUC2nd _term_/MIC ≥ 400 μg·h/mL	15/19 (78.9)	3/12 (25.0)	11.25 (2.03–62.20)	0.003	23.14 (2.31–431.53)	0.008
Achievement of source control	12/17 (70.6)	6/14 (42.9)	3.20 (19.72–14.15)	0.119	8.37 (0.86–81.38)	0.067
Comorbidity						
Charlson Comorbidity Index > 11 (median)	6/11 (54.5)	12/20 (60.0)	0.80 (0.18–3.54)	1.000		
Myocardial infarction/congestive heart failure	14/23 (60.9)	4/8 (50.0)	1.56 (0.31–7.85)	0.689		
Diabetes	11/19 (57.9)	7/12 (58.3)	0.98 (0.23–4.25)	0.981		
Liver disease	1/3 (33.3)	17/28 (60.7)	0.32 (0.03–4.01)	0.558		
Cerebrovascular disease	2/5 (40.0)	16/26 (61.5)	0.42 (0.06–2.95)	0.625		
Chronic pulmonary disease	3/5 (60.0)	15/26 (57.7)	1.10 (0.16–7.74)	1.000		
Any malignancy within 5 years	5/10 (50.0)	13/21 (61.9)	0.62 (0.13–2.82)	0.701		
Leukaemia/lymphoma	0/0	18/31 (58.1)	-	-		

Data are shown as n (%) for an early clinical response. SOFA = Sequential Organ Failure Assessment; OR = odds ratio; CI = confidence interval. * Secondary bacteraemia except for a central line-associated blood stream infection, ventilator associated pneumonia, thoracic empyema, pulmonary abscess, or osteoarthritis.

**Table 4:** The classification of the regimen according to the cumulative initial two doses (a loading dose and a maintenance dose) and AUC2nd _term_ in each dose regimen.

Classification of the Regimen According to the Cumulative Initial Two Doses	Vancomycin Dose (mg/kg)			AUC2nd _term_ (μg·h/mL)				
	Loading Dose	Maintenance Dose	Total Two Doses	Target Attainment (400–700)	<400	400–600	600–700	≥700
Low-dose regimen (n = 9)	20.5 ± 2.2	8.0 ± 1.1	28.5 ± 1.8	0.0%	100.0%	0.0%	0.0%	0.0%
Standard-dose regimen (n = 68)	26.7 ± 1.8	9.0 ± 0.9	35.8 ± 1.8	69.1%	30.9%	66.2%	2.9%	0.0%
High-dose regimen (n = 42)	30.7 ± 1.3	9.9 ± 0.7	40.6 ± 1.5	90.5%	9.5%	71.4%	19.0%	0.0%

Data are shown as the percentages for the vancomycin dose and the mean ± standard deviation for the AUC.

## Data Availability

The data presented in this study are available from the corresponding author on reasonable request.

## Supplementary Materials

The following supporting information can be downloaded at https://www.mdpi.com/article/10.3390/antibiotics15010047/s1, Table S1: HD-related factors; Table S2: Univariate and multivariate analyses of variables associated with an early clinical response in patients infected with resistant Gram-positive organisms; Table S3: Patients’ background; Table S4: Population pharmacokinetics model.

## Abbreviations

The following abbreviations are used in this manuscript:

AUC	Area under the curve
MIC	Minimum Inhibitory Concentration
HD	Haemodialysis
HDF	Haemodiafiltration
TDM	Therapeutic drug monitoring
PK	Pharmacokinetic
PD	Pharmacodynamic
