# Supplementary material for: Vancomycin Dosing Regimen to Obtain the Target Area Under the Concentration–Time Curve, Which Provides an Early Treatment Response for Patients on Haemodialysis
Source: Antibiotics (Basel). 2026 Jan 3;15(1):47. doi: 10.3390/antibiotics15010047 (PMC12838393; doi:10.3390/antibiotics15010047)
Supplement: Supplementary file 1 [file antibiotics-15-00047-s001.zip › antibiotics-4017777-supplementary.pdf]

Supplementary Materials

Table S1. HD-related factors

Variable	No. of patients (%) or the mean ± standard deviation
Haemodialyser	
Polysulfone	117 (98.3)
Clearance value for vitamin B12 (mL/min)	138.2±13.3
Clearance value for creatinine (mL/min)	189.9±2.6
Cellulose triacetate	2 (1.7)
Clearance value for vitamin B12 (mL/min)	163, 177
Clearance value for creatinine (mL/min)	197, 200
Membrane surface area	
1.1 m^2	38 (31.9)
1.5 m^2	48 (40.3)
1.8–1.9 m^2	19 (16.0)
2.1–2.5 m^2	14 (11.8)
Session time (h)	3.5±0.5
Blood flow rate (mL/min)	174.6±36.7
Dialysate flow rate (mL/min)	500.0±0.0
Ultrafiltration volume (mL/session)	1645.2±797.4
Predicted HD clearance of the unbound fraction (mL/min)	107.3±18.3
Time from initiating HD	
<3 months	25 (21.0)
3 months to 2 years	22 (18.5)
≥2 years	72 (60.5)
Inter-dialysis interval: 72 h	
Between the 1st and 2nd HD sessions	37 (31.1)
Between the 2nd and 3rd HD sessions	24 (20.2)

Table S2. Univariate and multivariate analyses of variables associated with an early clinical response in patients infected with resistant gram-positive organisms

Factor	No. of patients with an early clinical response (%)		Univariate analysis		Multivariate analysis	
	Patients with a particular factor	Patients without a particular factor	Crude OR (95% CI)	p value	Adjusted OR (95% CI)	p value
Male sex	22/31 (71.0)	13/19 (68.4)	1.13 (0.33–3.90)	0.849		
ICU stay	5/7 (71.4)	30/43 (69.8)	1.08 (0.19–6.32)	1.000		
Age ≥65 years	29/39 (74.4)	6/11 (54.5)	2.42 (0.60–9.68)	0.269		
Body mass index < 18.5 kg/m^2	7/11 (63.6)	28/39 (71.8)	0.69 (0.17–2.82)	0.713		
Body mass index ≥25 kg/m^2	7/11 (63.6)	28/39 (71.8)	0.69 (0.17–2.82)	0.713		
Surgery within 30 days	9/11 (81.8)	26/39 (66.7)	2.25 (0.42–11.96)	0.468		
Severity of illness						
SOFA score >5 (median)	17/24 (70.8)	18/26 (69.2)	1.08 (0.32–3.63)	0.902		
Septic shock	0/0	35/50 (70.0)	–	–		
Mechanical ventilation	2/4 (50.0%)	33/46 (71.7)	0.39 (0.05–3.10)	0.574		
Type of infection						
Complicated by MRSA infection*	17/28 (60.7)	18/22 (81.8)	0.34 (0.09–1.29)	0.106	0.14 (0.03–0.76)	0.042
Bloodstream infection	14/19 (73.7)	21/31 (67.7)	1.33 (0.37–4.74)	0.656		
Bone and joint infection	8/13 (61.5)	27/37 (73.0)	0.59 (0.16–2.25)	0.493		
Skin and soft tissue infection	6/9 (66.7)	29/41 (70.7)	0.83 (0.18–3.86)	1.000		
Respiratory tract infection	5/8 (62.5)	30/42 (71.4)	0.67 (0.14–3.24)	0.683		
Intra-abdominal infection	7/8 (87.5)	28/42 (66.7)	3.50 (0.39–31.32)	0.407		
Urinary tract infection	1/2 (50.0)	34/48 (70.8)	0.41 (0.02–7.05)	0.514		
Co-infection with gram-negative bacteria	15/21 (71.4)	20/29 (69.0)	1.13 (0.33–3.85)	0.851		
Co-infection with a fungal infection	2/2 (100.0)	33/48 (68.8)	–	1.000		
MRSA infection	18/31 (58.1)	17/19 (89.5)	0.16 (0.03–0.83)	0.019	0.18 (0.02–1.54)	0.118
$AUC_{2nd\ term}$/MIC ≥400 μg·h/mL	32/38 (84.2)	3/12 (25.0)	12.00 (2.87–50.21)	<0.001	24.04 (3.83–151.08)	0.001
Achievement of source control	23/29 (79.3)	12/21 (57.1)	2.88 (0.83–10.00)	0.091	5.25 (0.96–28.83)	0.056
Comorbidity						
Charlson Comorbidity Index >11 (median)	18/24 (75.0)	17/26 (65.4)	1.59 (0.47–5.42)	0.459		
Myocardial infarction/congestive heart failure	27/37 (73.0)	8/13 (61.5)	1.69 (0.45–6.40)	0.493		
Diabetes	19/28 (67.9)	16/22 (72.7)	0.79 (.023–2.70)	0.709		
Liver disease	4/6 (66.7)	31/44 (70.5)	0.84 (0.14–5.16)	1.000		
Cerebrovascular disease	5/8 (62.5)	30/42 (71.4)	0.67 (0.14–3.24)	0.683		
Chronic pulmonary disease	4/6 (66.7)	31/44 (70.5)	0.84 (0.14–5.16)	1.000		
Any malignancy within 5 years	10/15 (66.7)	25/35 (71.4)	0.80 (0.22–2.94)	0.747		
Leukaemia/lymphoma	2/2 (100.0)	33/48 (68.8)	–	1.000		

Data are shown as n (%) for an early clinical response. SOFA=Sequential Organ Failure Assessment; OR=odds ratio; CI=confidence interval.

*Secondary bacteraemia except for a central line-associated blood stream infection, ventilator associated pneumonia, thoracic empyema, pulmonary abscess, or osteoarthritis.

Table S3. Patients' background

Variable	Patients on haemodialysis (n=119)	Patients not on haemodialysis (n=112)	p value
Male sex, n (%)	75 (63.0)	70 (62.5)	0.934
Age (years)	71.6±13.5	69.1±14.0	0.165
Body weight (kg)	54.8±11.0	56.6±14.6	0.283
Body mass index (kg/m^2)	22.9±14.1	21.5±4.7	0.331
Creatinine clearance (mL/min)	–	93.7±35.2	
ICU stay, n (%)	11 (9.2)	13 (11.6)	0.556
Surgery within 30 days, n (%)	19 (16.0)	26 (23.2)	0.103
Comorbidities			
Charlson Comorbidity Index	10.4±2.7	6.0±3.6	<0.001
Ischaemic heart disease, n (%)	62 (52.1)	14 (12.5)	<0.001
Congestive heart failure, n (%)	54 (45.4)	18 (16.1)	<0.001
Peripheral vascular disease, n (%)	48 (40.3)	4 (3.6)	<0.001
Dementia, n (%)	8 (6.7)	4 (3.6)	0.281
Cerebrovascular disease, n (%)	23 (19.3)	24 (21.4)	0.692
Chronic pulmonary disease, n (%)	19 (16.0)	7 (6.3)	0.020
Collagen vascular disease, n (%)	26 (21.8)	8 (7.1)	0.002
Gastrointestinal disease, n (%)	50 (42.0)	15 (13.4)	<0.001
Paralysis, n (%)	9 (7.6)	6 (5.4)	0.497
Diabetes without end-organ damage, n (%)	5 (4.2)	30 (26.8)	<0.001
Diabetes with end-organ damage, n (%)	56 (47.1)	0 (0.0)	<0.001
Moderate or severe renal disease, n (%)	119 (100.0)	0 (0.0)	<0.001
Urine output ≥0.5 mL/kg/h, n (%)	3 (2.5)	112 (100.0)	<0.001
Mild liver disease, n (%)	10 (8.4)	7 (6.3)	0.531
Moderate or severe liver disease, n (%)	8 (6.7)	2 (1.8)	0.103
Any malignancy within 5 years, n (%)	41 (34.5)	28 (25.0)	0.117
Metastatic solid tumour, n (%)	3 (2.5)	16 (14.3)	0.001
Leukaemia, n (%)	3 (2.5)	1 (0.9)	0.622
Lymphoma, n (%)	6 (5.0)	4 (3.6)	0.750
AIDS/HIV, n (%)	0 (0.0)	0 (0.0)	–
Severity			
Mechanical ventilation, n (%)	9 (7.6)	11 (9.8)	0.542
Sequential Organ Failure Assessment score when 4 points for renal factors were excluded	2.8±2.5	1.7±2.6	<0.001

Data are shown as n (%) for an early clinical response and as the mean ± standard deviation for other data. ICU= Intensive Care Unit

Table S4. Population pharmacokinetics model

Parameter	Estimated value (%RSE) [%shrinkage]
Fixed-effect parameters	
CL =θ1×(body weight/70) 0.75×exp(η1) unbound fraction×KoA-predicted CL of HD if (during HD) 1 else 0	
θ1	0.316 (8.5%)
VSS=θ2×body weight×EXP(η2)	
θ2	1.160 (7.0%)
K12=θ3	
θ3 (K12)	0.525, fixed
K21=θ4×EXP(η3)	
θ4 (K21)	0.213, fixed
Random-effect parameters	
Interindividual variability ω1 (of CL)	0.365 (36.2%) [11.8%]
Interindividual variability ω2 (of VSS)	0.302 (34.1%) [9.2%]
Interindividual variabilityω3 (of K21)	0.286, fixed
Residual variability (σ)	0.064 (15.7%)

RSE=relative standard error; CL=clearance; HD=haemodialysis; VSS=volume of distribution at steady state; K12=transfer constant from the central to the peripheral compartments; K21=transfer constant from the peripheral to the central compartments. The KoA-predicted clearance during HD was calculated using plasma and dialysate flow rates, the ultrafiltration rate, and haemodialyser characteristics.
